# Bridging expectations and science: a roadmap for the future of longevity interventions

**DOI:** 10.1007/s10522-025-10278-z

**Published:** 2025-07-01

**Authors:** Danyang Yu, Xinyi Zeng, David Barzilai, Dominik Thor, Yu-Xuan Lyu

**Affiliations:** 1 Sirio Institute for Anti-Ageing (SIA), Sirio Life Technology Co., Ltd, Shanghai, 201703 China; 2 Geneva College of Longevity Science, 1204 Geneva, Switzerland; 3 Healthspan Coaching LLC, Barzilai Longevity Consulting, Boston, MA 02111 USA; 4 Harvard Medical School, Boston, MA 02115, USA; 5 Institute of Advanced Biotechnology, Institute of Homeostatic Medicine, and School of Medicine, Southern University of Science and Technology, Shenzhen, 518055 China

**Keywords:** Longevity intervention, Public perception, Healthspan, Translational science, Roadmap

## Abstract

The field of longevity interventions has witnessed rapid expansion, driven by scientific advancements alongside growing industry and consumer interest. However, no longevity intervention has yet been proven effective or ready for widespread clinical adoption. A substantial gap persists between public expectations and the current scientific realities. This article explores four key themes: (1) consumer priorities regarding longevity interventions, (2) the type and depth of scientific information they value, (3) psychological, financial, and practical barriers limiting adoption, and (4) potential strategies to overcome these challenges. Despite increasing enthusiasm, clinical translation of longevity research is constrained by the lack of validated interventions, regulatory frameworks, and standardized biomarkers. By distinguishing between scientifically supported and unproven approaches, this article proposes a roadmap outlining the critical milestones necessary to advance longevity interventions from research to clinical readiness. The goal is to realign public understanding with the current state of longevity science and guide future efforts toward safe and effective translation.

## Introduction

Over the past decades, research on ageing has progressed substantially, unveiling critical biological mechanisms that drive ageing and informing the development of interventions aimed at prolonging not only lifespan but also healthspan (Campisi et al. 2019; Guo et al. 2022; Lyu et al. 2024). This progress has fueled substantial growth in the longevity industry, propelled by innovations in biotechnology and growing consumer demand for interventions that promote healthspan extension. Longevity interventions encompass a broad spectrum of strategies, including lifestyle modifications, pharmacological therapies, and emerging biotechnologies, all aimed at targeting fundamental ageing mechanisms, such as cellular senescence, epigenetic alterations, and mitochondrial dysfunction. The global complementary and alternative medicine market for anti-ageing and longevity was valued at approximately $63.6 billion in 2023 and is projected to grow at a compound annual growth rate (CAGR) of 21.5% from 2024 to 2030 (Grand View Research 2023).

Despite this commercial surge, relatively few interventions have demonstrated clinical benefits in human populations, a challenge partly attributed to the intrinsic heterogeneity of ageing (Gonzalez-Freire et al. 2020). Concurrently, academic interest has intensified; a bibliometric analysis of Web of Science reveals that publications containing “anti-ageing” or “longevity” keywords increased from 32,843 during 1991–2015 to 35,399 in the 2016–2024 period. Moreover, leading scientific publishers such as *The Lancet* and *Springer Nature* have launched special journals, *The Lancet Healthy Longevity* and *Nature Aging*, with impact factors of 13.4 and 17.0 in 2023, respectively.

However, despite the growing volume of publications, translational progress remains limited due to the scarcity of validated therapeutic options available to consumers. This article explores several key dimensions, including consumer priorities and expectations regarding longevity interventions, the challenges in effectively communicating scientific evidence to the public, and the psychological, financial, and regulatory barriers that limit equitable access to safe and effective solutions. By synthesizing these perspectives, the article underscores the urgent need to bridge public expectations and scientific realities, and proposes a roadmap to foster the development of comprehensive and accessible strategies for extending healthspan.

### What do consumers want? (consumers demand)

#### Healthspan vs lifespan extension

To avoid conceptual ambiguity, we define several closely related terms central to this field, such as lifespan, healthspan, longevity and anti-ageing (Table 1). According to an empirical study, the assurance of sustained health significantly shapes public acceptance of lifespan extension. When respondents were assured of continued physical and mental health, 79.7% expressed a desire for lifespans exceeding 120 years, and 53.1% opting for indefinite longevity. In contrast, without health guarantees, 65.3% of participants preferred limiting their lifespan to 85 years (Donner et al. 2016). This finding suggests that consumers prioritize healthspan, especially the maintenance of physiological and cognitive vitality, before seeking lifespan extension. Notably, respondents with scientific interest were substantially more likely to prioritize healthspan and shifted their preferences towards lifespans exceeding 120 years when health was guaranteed. This indicates that scientifically engaged individuals better understand healthspan as an essential prerequisite for desiring extended lifespan, rather than simply endorsing indefinite longevity.

**Table 1:** Glossary of terms

Term	Definition	Refs
Lifespan	Total time an individual lives, from birth to death	Dong et al. 2016
Healthspan	The portion of life spent in good health, free from serious chronic diseases or disability, with preserved physical and cognitive functions	Kaeberlein 2018
Longevity	The ability to live significantly beyond the average life expectancy under optimal conditions	De Benedictis & Franceschi 2006
Anti-ageing	Biomedical or lifestyle strategies aimed at delaying, preventing, or partially reversing physiological and functional decline associated with ageing	Ok 2022

However, the high proportion of individuals endorsing longevity under ideal health conditions may reflect an optimistic belief in the possibility of radical lifespan extension. This optimism could imply a complex relationship between scientific interests and acceptance of health-guaranteed lifespan extension: while some individuals with strong scientific interests might embrace the theoretical potential for extreme longevity, others remain more cautious (Donner et al. 2016). Explicitly acknowledging this complexity helps align public expectations with the current scientific realities and highlights the critical role of transparent science communication in managing optimism and skepticism regarding longevity advancements. These insights suggest that emphasizing the centrality of healthspan, rather than lifespan extension alone, is crucial for effectively communicating longevity science and managing public expectations.

#### Practical outcomes vs theoretical advancements

Ageing biomarkers are often hypothesized as biological indicators that could objectively reflect biological age, predict age-related diseases, and evaluate longevity interventions. However, this assumption remains largely unfulfilled in clinical practice (Moqri et al. 2024). Many studies remain experimental or concentrate on these long-term ageing biomarkers, like epigenetic clocks or telomere elongation (López-Otín et al. 2023; Kennedy et al. 2014). However, despite their scientific promise, biological clocks often show variability between individuals and across tissues, and their sensitivity to short-term interventions remains uncertain. Meanwhile, based on our empirical observations of the nutraceutical industry, consumers tend to prioritize tangible health benefits, such as improved physical function, reduced disease risk, or a more youthful appearance, over abstract changes in ageing biomarkers. Since most ageing biomarkers, including epigenetic clocks and telomere length, lack interventional reversibility data, none are currently validated as clinical endpoints for guiding therapeutic decisions. The longevity intervention consumers want something much more tangible and distinct: an improved quality of life. While biomarkers hold scientific value for measuring biological age, their clinical translation requires clear linkages to actionable outcomes. For example, interventions validated by biomarkers must demonstrate measurable improvements in healthspan metrics like mobility, cognitive vitality, or disease resilience to gain consumer trust.

#### Demographics variations

Age, gender and culture significantly shape attitudes toward longevity and anti-ageing research and interventions. For example, younger adults (18–29 years) prioritize aesthetic preservation, preferring to halt ageing at a mean age of 23.08 (Barnett & Helphrey 2021). In contrast, older adults (60 + years) focus on healthspan extension to mitigate age-related diseases. This study reveals distinctions even within older cohorts: younger-old (60–84) and older-old (85 +) respondents selected mean indefinite ages of 69.12 and 77.07, respectively, suggesting a gradual recalibration of expectations with advancing age.

Gender differences further complicate adoption patterns. Men exhibit a 1.5 times greater willingness to adopt life-extension therapies, while women dominate cosmetic anti-ageing markets, reflecting divergent societal norms (Barnett & Helphrey 2021). Another research study also shows similar results on gender differences in attitudes towards longevity interventions, with males being more supportive of life-extension research and more likely to use a potential life-extension technology than females (Partridge et al. 2011). These differences highlight the need for age- and gender-specific interventions that balance aesthetic aspirations with functional health outcomes. Aesthetic goals may reflect cultural or age-based values; however, these remain largely uncorrelated with validated healthspan metrics.

Cultural values and perceptions profoundly influence attitudes towards longevity. The term “anti-ageing” is widely used in public discourse, product labelling, and commercial branding. Despite its popularity, however, this term is sometimes met with skepticism within the scientific community, particularly in Western contexts. To distance their work from the pseudoscientific claims often associated with commercial industries, some researchers now prefer terms such as “geroscience” or “longevity medicine” (Le Couteur and Barzilai 2022; Bischof et al. 2021). In contrast, Asian professionals and markets tend to embrace the “anti-ageing” terminology. For example, in Japan, the direct translation of “longevity” (長寿, chōju) carries connotations of passive acceptance of ageing, whereas “anti-ageing” (アンチエイジング) is perceived as proactive and aspirational (Hidekazu Yamada 2024). Similar trends are also observed in China and other Asian countries, where “longevity” is typically linked to lifespan and historical aspirations of immortality, while “anti-ageing” (抗衰, Kang Shuai) refers to a more proactive approach focused on interventions to extend healthy life (Giulia Interesse 2024).

These cultural variations are also reflected in market practices. In Western countries, particularly the U.S. and Europe, “healthy ageing” and “successful ageing” are dominant paradigms that emphasize individual responsibility for health. This discourse supports the growth of anti-ageing medicine and consumer markets, yet also raises concerns about the commercialization of science, public understanding, and the control over health-related knowledge (Cardona 2008). In countries like Australia, the anti-ageing industry incorporates global trends inspired by the U.S. but adapts them to local cultural values and regulatory frameworks, resulting in hybridized and context-specific practices (Cardona 2009). This indicates that beyond linguistic preferences, the interpretation and implementation of “anti-ageing” strategies are deeply influenced by socio-cultural and institutional environments.

#### Conventional interventions vs radical interventions

A study found that public acceptance of longevity interventions is highly correlated with perceived safety and potential side effects. Research indicates that the acceptance rates are significantly higher for exercise (66%) and dietary supplements (82%) compared to pharmacological options like metformin (26%) and rapamycin (10%). This public skepticism toward pharmacologic interventions reflects not only risk aversion but also the absence of long-term randomized controlled trials (RCTs)-level evidence in healthy ageing populations (Brouwers et al. 2024). Consequently, more radical interventions are often excluded from the general public. One qualitative socio-empirical research highlighted that individuals often begin with lifestyle-based strategies and gradually transition to more intensive interventions as perceived risks are reassessed (Schweda and Pfaller 2014). Another review article on longevity interventions also suggested that the non-invasive interventions, such as exercise, intermittent fasting and antioxidants are safer, while more experimental approaches like stem cell therapy or plasma exchange require more critical assessments to determine their long-term efficacy and adverse effects (Shetty et al. 2018). This uncertainty and fear of adverse effects drive consumers to choose low-risk and preventive interventions.

These preferences are not only shaped by safety perception, but also deeply influenced by cultural interpretations of ageing and healthcare. For example, a comparative study found distinct initiation pathways for anti-ageing product use among elderly populations in Australia and Japan. Australian older adults are more likely to begin using supplements under medical advice, or alternatively, turn to self-directed use when dissatisfied with their doctors’ recommendations. In contrast, Japanese older adults tend to view minor health complaints as part of the natural ageing process and consider supplements as an extension of traditional “shokuji-ryōhō” (dietary therapy)—a culturally embedded practice of managing health through food. However, both groups demonstrate a shared resistance to radical medical interventions, particularly invasive procedures such as surgery. Their use of anti-ageing products and supplements is often motivated by a desire to delay or avoid such interventions altogether (Omori and Dempsey 2018).

Despite the popularity of supplements among consumers, it is important to note that most commercially available products marked as “anti-ageing” or “longevity” interventions still lack rigorous, longterm human trials that demonstrate their efficacy on validated healthspan outcomes and their safety. A striking example is the 2024 Beni-Koji scandal in Japan, where red yeast rice supplements produced by Kobayashi Pharmaceutical were linked to approximately 3,000 adverse health events, including 212 hospitalizations and five deaths due to acute renal failure resembling Fanconi syndrome (Hashimoto et al. 2024). Such food safety incidents remind us of an imperative principle: the economic gains derived from health-related products must never take precedence over safety considerations.

### Barriers and concerns

Despite growing interest, systemic barriers hinder the widespread adoption of longevity interventions.

#### Psychological barriers

Consumer skepticism persists due to historical overpromises in anti-ageing medicine. The credibility of longevity science has also been eroded by widespread commercialization of unvalidated supplements and therapies, contributing to public confusion and mistrust. For example, unregulated “anti-ageing” or “longevity” supplements marketed with exaggerated claims have eroded confidence in emerging therapies. Some scientists have also raised concerns about the limitations of translating findings from animal studies to human clinical trials, noting that the effects of pharmaceutical solutions are often overstated (Le Bourg 2022). The commercialization of longevity interventions may also exacerbate health inequities, raising ethical concerns about accessibility and resource allocation (Stambler 2018). Moreover, perceived benefits from these interventions may stem from placebo effects or increased health engagement rather than mechanistic efficacy, underscoring the critical need for rigorous controlled trials.

#### Financial constraints

High costs remain a significant barrier to the accessibility of longevity medicine, particularly for cutting-edge interventions. For example, Casgevy, the first CRISPR therapy approved by the FDA, is priced at $2.2 million per treatment, while its competitor Lyfgenia, a gene therapy for sickle cell disease, is listed at $3.1 million (Reuters 2023). These treatments are generally targeting rare conditions or narrowly defined clinical uses, making them inaccessible for broader preventive or anti-ageing applications among the general population.

GLP-1 receptor agonists such as semaglutide and liraglutide have recently gained prominence in the anti-ageing space. Originally developed for type 2 diabetes, these drugs are now widely used for obesity management and show potential longevity benefits, including improvements in mitochondrial function and reductions in chronic inflammation (Chavda et al. 2024; Peng et al. 2022). However, they remain costly, several hundred dollars per month (Wen et al. 2025), and insurance coverage is inconsistent. For example, Medicare in the U.S. does not cover GLP-1 s for weight loss, and while some are reimbursed in China, non-diabetic uses often require out-of-pocket payment. Long-term safety concerns include nausea, gastrointestinal discomfort, and potential receptor desensitization (Shetty et al. 2022; Kupnicka et al. 2024). The growing use of GLP-1 s for cosmetic or lifestyle purposes has also sparked debate about equitable access and the diversion of medical resources from patients with genuine clinical needs.

At the more affordable end of the spectrum are drugs like rapamycin and metformin, which have demonstrated promising results in preclinical models and exhibit a relatively safe profile in human populations (Moel et al. 2025; Barzilai et al. 2016). Rapamycin costs roughly $2.30 per tablet, amounting to about $100 per month, while metformin is even cheaper, with monthly costs as low as $13.72. Despite their low cost and broad availability, these drugs, like all other potential longevity interventions, are used off-label for ageing-related purposes and have not received regulatory approval for such indications. Moreover, they are not recognized by most national health insurance schemes or private insurers, requiring patients to pay out of pocket.

Beyond interventions, financial barriers are also evident in the broader field of longevity-focused healthcare services. Currently, longevity programmes offered by high-end clinics typically cost tens of thousands of U.S. dollars annually, depending on the range of diagnostic tests (e.g., whole genome sequencing, epigenetic clocks, VO₂ max testing, DEXA scans) and frequency of clinical consultations (The New York Times 2025a). This pricing structure creates a distinct stratification in access, where early-stage longevity science primarily benefits affluent individuals, while the vast majority are excluded due to cost. Most services are paid for privately, with only limited components partially reimbursed through commercial insurance.

#### Practical barriers

The development and implementation of longevity interventions face a range of technical, ethical, and regulatory challenges. For example, interventions, like supplements, drugs, gene therapy, stem cell therapy and stem cell-derived exosome treatments, remain limited by the lack of robust human clinical trials, despite some notable research progress demonstrating their therapeutic potential. Furthermore, successful translation into clinical practice necessitates addressing critical issues, including scalable manufacturing, standardized purification processes, and batch-to-batch consistency. These challenges are especially pronounced given the nature of complexity like stem cells and exosomes (Yin et al. 2019; Zhang et al. 2023).

Beyond technical limitations, substantial disparities in global regulatory frameworks further complicate the path to clinical adoption. Countries vary significantly in their capacity and readiness to adapt to ageing-related healthcare demands. For example, Nordic countries such as Norway and Sweden rank high in multidimensional societal adaptation, while Central and Eastern European nations lag behind. Even within a single country, different policy domains, such as productivity, welfare, and health security, may be unevenly developed (Chen et al. 2018). These differences extend to regulatory stances on longevity interventions.

In the European Union, the European Medicines Agency (EMA) maintains strict evidentiary standards for approving anti-ageing drugs, requiring robust clinical trial designs and long-term outcome validation. This has slowed the approval of many novel therapies, especially those targeting ageing as a biological process (Penella 2024a, b). In contrast, countries with more permissive regulatory environments, such as Australia, have allowed treatments like stem cell therapy to enter the market more easily (Cardona 2009). While this flexibility may accelerate innovation, it also increases the risk of premature commercialization in the absence of rigorous oversight.

Meanwhile, regulatory ambiguity and inconsistent enforcement in many regions have enabled the proliferation of unverified “anti-ageing” or “longevity” products and therapies, creating a fragmented and misleading consumer landscape. This contributes to a “promise-performance gap,” where exaggerated marketing claims outpace clinical validation, eroding public trust. Moreover, current public health communication systems and market regulations in many countries remain ill-equipped to protect consumers from misinformation and exploitation (Mehlman et al. 2004).

### The communication gaps

Bridging the gap between consumer expectations and the realities of complex scientific endeavours, like the search for reliable biomarkers of ageing, requires clear communication between scientists and the public. This is particularly true in the area of public health, which often intersects with the expectations that arise from scientific work.

#### Levels of detail appreciated

Based on our empirical observations and internal stakeholder interviews, the levels of detail consumers appreciated are highly correlated with ageing biomarkers, ranging from molecular indicators (e.g., telomere length, epigenetic changes) to organ- or function-specific outcomes (e.g., cardiovascular health, cognitive decline). Consumers prefer tangible health benefits to abstract ageing biomarkers; in the meantime, they also appreciate some simple, straightforward, forward, visible readouts to explain the health outcomes. However, the development and application of ageing biomarkers are still facing four key challenges: (1) Standardized biological age definitions are lacking, with inconsistent use of chronological age, mortality risk, or proxy phenomenal data; (2) Data source reproducibility and technical limitation variability, where molecular, imaging, and clinical biomarkers differ; (3) Biomarker models exhibit a “prediction-association paradox” where improved chronological age prediction may diminish their biological relevance to ageing phenotypes, such as mortality. (4) For biomarkers to have clinical utility, they must undergo rigorous longitudinal validation to confirm that changes are predictive of and responsive to interventions affecting age-related disease trajectories. (Chen et al. 2023; Zhang et al. 2019).

#### Miscommunication in longevity science outreach

The challenge lies in translating complex scientific findings into accessible, actionable insights. Miscommunication often arises from technical jargon, overhyped claims, or insufficient explanation of research limitations, leading to unrealistic consumer expectations. For example, studies highlight the potential misinterpretation or misuse of DNA methylation ageing clocks might foster misconceptions about their immediate clinical utility and cause social anxiety. Yet those clocks only have limited evidence base when it comes to identifying actionable interventions. (Bell et al. 2019).

Prominent figures in the biohacking space often simplify complex interventions for public engagement. While this increases visibility, it can unintentionally obscure scientific uncertainty and overstate efficacy. Moreover, this over-simplified information might neglect the long-term safety issues and overstate the efficacy of these interventions. The public might be misled into unrealistic expectations without a comprehensive and accurate portrayal of scientific evidence. For example, some commercialized approaches to longevity interventions have faced criticism for being potentially unsustainable, expensive, and lacking rigorous scientific and clinical data support (The New York Times 2025b). This model of longevity science outreach prioritizes actionable claims over transparent disclosure of scientific uncertainties. Moreover, its scientific communication is driven by commercial purposes and might create a public misperception of “scientifically packaged” claims as evidence-based consensus. Certain commercial entities strategically adopt the terminology of anti-ageing or longevity medicine to position consumer products or compound classes, often without clinically validated efficacy, as part of the evidence-based longevity landscape, thereby leveraging the field’s scientific credibility for marketing purposes.

### Regulations and standards

#### Regulatory gaps

The longevity sector currently exists in an environment of regulatory ambiguity. Many emerging interventions, such as biological age diagnostics, senolytic compounds, and longevity-focused wellness clinics, often operate in advance of regulatory frameworks still under development, particularly when used for wellness optimization, off-label purposes, or within biohacker communities. These uses typically fall outside the scope of traditional regulatory oversight, not due to illegality or unethical intent, but because no clear approval pathways yet exist for interventions targeting ageing itself.

To clarify, while some unapproved therapies are being explored in fragmented and non-clinical settings, they lack robust validation and standardized oversight. Rather than signaling readiness, these activities highlight the urgent need to establish a structured roadmap for responsible translation. Defining regulatory criteria, validating biomarkers, and developing evidence-based endpoints are critical foundational steps for any future longevity intervention to move toward clinical viability.

Despite their growing visibility, ageing biomarkers have not been qualified by agencies such as the FDA or EMA, primarily due to the absence of longitudinal validation studies linking them to clinical outcomes. A major obstacle is the lack of consensus on whether ageing should be recognized as a modifiable condition, even though we know it is highly associated with lots of pathologically defined conditions like sarcopenia, osteoporosis, and several neurodegenerative disorders. This has historically constrained drug development and trial design (The Lancet Healthy Longevity 2022). The FDA’s tentative shift toward recognizing ageing as a modifiable condition, exemplified by its consideration of the Targeting Aging with Metformin (TAME) trial, a landmark clinical study designed to test metformin’s ability to delay multiple age-related diseases collectively (Vaiserman and Lushchak 2017; Barzilai et al. 2016).

#### FAIR standards and biomarker evaluation

Establishing standardized guidelines and rigorous evaluation criteria is crucial to ensure consumer safety and build trust. A paradigm shift is needed in the validation of ageing biomarkers, moving toward standardized, collaborative frameworks. Global guidelines must enforce FAIR (Findable, Accessible, Interoperable, Reusable) data principles and emphasize clinical relevance over commercial hype. Adopting FAIR practices and promoting transparent reporting, including null or negative results, is essential to prevent cherry-picking of favourable data and premature commercialization. Stakeholders must ensure that biomarkers, such as epigenetic clocks, proteomic clocks, and transcriptomic clocks, become safe and effective tools for measuring public healthspan (Moqri et al. 2024). Moreover, a centralized registry of negative or inconclusive longevity trials could reduce duplication and curb misleading narratives driven by selective publication.

### Strategies for bridging the gap

#### Holistic vs reductionist interventions

To overcome consumers’ psychological barriers to longevity interventions, a scientifically grounded approach should prioritize multi-dimensional strategies consistent with homeodynamic principles. Emphasizing holistic practices, such as regular physical activity, dietary modulation, and cognitive-social engagement over single-molecule therapies can help mitigate skepticism rooted in historical overpromises. Evidence supports lifestyle interventions as foundational strategies for healthspan extension, and these should not be conflated with the lower evidence claims often associated with many supplements or isolated molecule therapies.

Transparent communication of evidence-based benefits and candid discussions of limitations and ethical considerations are critical to foster trust (Rattan 2020). Moreover, avoiding exaggerated claims is vital to prevent public disillusionment and uphold scientific integrity (Aparicio 2025). A clear hierarchy of evidence should guide both public understanding and policy, prioritizing interventions supported by randomized controlled trials over those based solely on molecular or preclinical indicators.

#### Biomarker-linked clinical application

Addressing consumer demand for tangible benefits while advancing ageing biomarker research requires linking biomarkers to actionable interventions through rigorous clinical and technical validation (Moqri et al. 2024). Progress requires establishing a clear association between biomarkers and meaningful health outcomes through well-powered, prospective clinical studies rather than relying solely on correlative or surrogate endpoint data. However, significant challenges persist in developing universally predictive biomarkers for individual lifespan or healthspan due to the multidimensional nature of ageing and considerable inter-individual heterogeneity (López-Otín et al. 2023). Although composite biomarkers show promise for population-level predictions, their effectiveness for personalized longevity interventions remains under investigation. Ensuring affordability by developing cost-effective treatments, diagnostic devices, and measurement tools is paramount. Promoting insurance coverage for such biomarker measurements can also enhance accessibility. Effective collaboration among researchers, clinicians, policymakers, and other stakeholders is vital to align scientific innovations with real-world needs and expectations (Biomarkers of Aging Consortium 2024, and Lyu et al. 2024).

#### Global standardization of ageing biomarkers

Global standardization of ageing biomarkers is essential for clinical translation. However, no biomarker has yet been formally qualified as a surrogate endpoint by regulatory agencies like the FDA, mainly due to methodological inconsistencies and poor cross-population generalizability (Moqri et al. 2023). To address this, initiatives such as the Aging Biomarker Consortium (ABC) and Biomarkers of Aging Consortium (BAC) aim to establish shared validation standards (Aging Biomarker Consortium 2023).

Existing international mechanisms offer valuable models. The World Health Organization (WHO) promotes global consensus via expert panels, while the International Council for Harmonisation of Technical Requirements for Pharmaceuticals for Human Use (ICH) provides harmonized regulatory guidelines, such as E6 Good Clinical Practice (European Medicines Agency 2016) and E9 Statistical Principles for Clinical Trials (Lewis 1999). The Organisation for Economic Co-operation and Development (OECD)’s three-stage biomarker validation model—covering analytical validity, clinical relevance, and utility—can also inform ageing biomarker evaluation (OECD 2013).

A coordinated effort should involve regulators (FDA, EMA, PMDA), global health bodies (WHO, ICH), research consortia, and industry. These stakeholders can collaborate through expert consensus (e.g., Delphi method) (Perri et al. 2025), shared databases, and public–private partnerships. A phased approach is recommended: (1) Short-term: Consensus building and data harmonization; (2) Mid-term: Validation studies and regulatory engagement; (3) Long-term: Integration into clinical trials and formal qualification.

#### Scientific communication

Bridging communication gaps necessitates collaboration between researchers and patient advocates to develop accessible educational programmes and leverage social media platforms for disseminating balanced, evidence-based information that translates ageing research into practical interventions. A Europe-wide survey revealed that only 16–20% of citizens regard mass media as the preferred channel for communicating scientific societal impacts, whereas 63% favour direct communication from researchers (González Pedraz 2018). Rather than reactively addressing misinformation, researchers should proactively partner with public communicators to co-create accurate, nuanced explanations of longevity science. Utilizing social media for bidirectional engagement, such as live Q&A sessions or data interpretation tutorials, can enhance public understanding of the scientific uncertainties inherent in longevity interventions. However, this is effective only when the medium’s limitations, including users’ reduced attention spans and preference for brief content, are acknowledged and proactively addressed. Research indicates that the rapid consumption patterns fostered by social media platforms can diminish users’ capacity for sustained attention, necessitating tailored communication strategies to maintain engagement with complex health information (Chiossi et al 2023).

#### Regulatory guidelines

Clear and consistent regulatory guidelines are critical to bringing longevity interventions safely into clinical practice. Currently, progress is hindered by the lack of a globally accepted definition of ageing and the absence of validated surrogate endpoints. Establishing agreement on clinically relevant outcomes, such as frailty progression, functional decline, or molecular markers, would provide a solid foundation for trial design and regulatory evaluation.

To move forward, existing international frameworks like the ICH and WHO can serve as practical starting points. The TAME trial, which adopts functional endpoints to evaluate metformin’s effect on ageing-related diseases, offers a useful model. Building on this, a dedicated international task force, similar to ICH working groups, could help align standards for ageing biomarkers, outcome measures, and ethical oversight.

This effort should involve collaboration among regulators (e.g., FDA, EMA, PMDA), research consortia (e.g., BAC, Geroscience Network), global health organizations (e.g., WHO, OECD), and industry stakeholders. Jointly, they can develop guidelines for ageing-related interventions and promote trial transparency by integrating ageing studies into national systems and global registries like the WHO ICTRP. Creating such pathways will help ensure that new interventions meet high standards for safety and efficacy, while also accelerating access to innovation in the field of longevity science.

## Conclusion

This article highlights the critical need to align anti-ageing and longevity research with consumer priorities to enhance both adoption and real-world impact. Given the current evidence, it is fair to say that longevity interventions are not yet “ready” for widespread clinical or consumer use. Achieving this readiness requires a multifaceted approach, which we propose as a roadmap comprising several key milestones:

1. Systematically mapping public attitudes toward evidence-based interventions to better guide clinical translation efforts.
2. Fostering cross-sector collaborations to establish global standards for ageing biomarkers alongside coherent regulatory frameworks.
3. Reducing the cost of interventions to improve accessibility and equity.
4. Prioritizing authentic education and transparent scientific communication to combat misinformation and prevent consumer disillusionment.

**Fig. 1:**
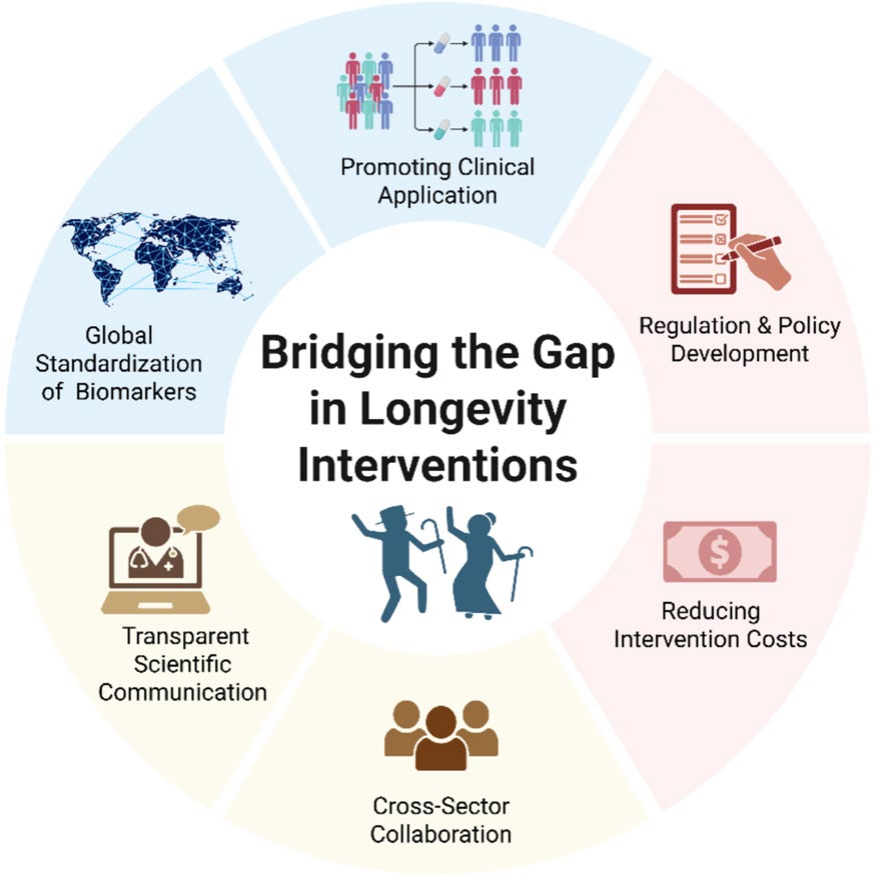
Strategy Roadmap for Bridging the Gap Between Public Expectations and Scientific Realities in Longevity Interventions. The scheme of strategy roadmap for bridging gaps proposed in this work: promoting clinical application, regulation and policy development, reducing intervention costs, cross-sector collaboration, transparent scientific communication and global standardization of biomarkers

Together, these steps will help bridge the gap between scientific advances and consumer needs, ultimately paving the way for more effective, accessible, and consumer-centered longevity solutions that address the complexities of ageing while fostering trust and sustained engagement (see Fig. 1).

### Limitations of this study

This manuscript represents a synthesis of current knowledge on public perceptions of longevity interventions, but several limitations warrant acknowledgement. First, the field lacks robust, large-scale empirical studies on consumer priorities, requiring us to rely on fragmented data sources such as cross-sectional surveys. Second, although we have strived for objectivity, our interpretation of consumer attitudes and regulatory challenges may reflect inherent biases in available literature, which disproportionately focuses on Western markets. Finally, our discussion of intervention feasibility assumes relatively linear progress in scientific and regulatory frameworks, potentially overlooking systemic barriers like funding inequities or political constraints. These limitations underscore the urgent need for more interdisciplinary research to build a comprehensive and realistic foundation for future advancements in the field.

## Data Availability

No datasets were generated or analysed during the current study.
